# Switching between Multiple Codes of SNARC-Like Associations: Two Conceptual Replication Attempts with Anodal tDCS in Sham-Controlled Cross-Over Design

**DOI:** 10.3389/fnins.2017.00654

**Published:** 2017-11-23

**Authors:** Philipp A. Schroeder, Hans-Christoph Nuerk, Christian Plewnia

**Affiliations:** ^1^Department of Psychiatry and Psychotherapy, Neurophysiology and Interventional Neuropsychiatry, University of Tübingen, Tübingen, Germany; ^2^Department of Psychology, Diagnostics and Cognitive Neuropsychology, University of Tübingen, Tübingen, Germany; ^3^Leibniz-Institut für Wissensmedien, Tübingen, Germany; ^4^LEAD Graduate School & Research Network, University of Tübingen, Tübingen, Germany; ^5^Werner Reichardt Centre for Integrative Neuroscience, Tübingen, Germany

**Keywords:** prefrontal cortex, (non-)numerical cognition, transcranial direct current stimulation, SNARC effect, ordinal sequence

## Abstract

In societies with left-to-right reading direction, left-side vs. right-side behavioral decisions are faster for relatively small vs. large number magnitudes, and vice versa, a phenomenon termed Spatial-Numerical Associations of Response Codes (SNARC) effect. But also for non-numerical sequential items, SNARC-like effects were observed, suggesting a common neurocognitive mechanism based on the ordinal structures of both numbers and sequences. Modulation of prefrontal networks that are involved in providing spatial associations during cognitive behavior can contribute to elaborate their neuropsychological theoretical foundations. With transcranial direct current stimulation (tDCS) directed to the left prefrontal cortex, we recently showed that (i) cathodal tDCS can block the emergence of spatial-numerical associations and that (ii) anodal tDCS can reverse spatial associations of sequential order, most likely based on markedness correspondence. Two conceptual replication attempts of the latter reversal of space-order associations are presented in the current sham-controlled experiment, using either weekdays (Monday-Friday) or month names (January-December) as stimuli in the temporal order classification task. In addition, to control for possible influences of notation, number stimuli were presented as written German names (One-Five). We report on a successful modulation of spatial-numerical associations of response codes (SNARC) effects with month stimuli induced by anodal tDCS, but failed to observe the same reversal of SNARC effects for weekday stimuli. The former stimulation effect was orthogonal to the small anodal tDCS effect on written number words, which replicates the dissociation of SNARC effects for numbers vs. non-numerical sequences. Moreover, this result reinforces the hypothesis that the ordinal item and task structure was the source of dissociation (as opposed to verbal presentation). We suggest that the diverging results can be explained by the markedness correspondence account of spatial associations in a multiple coding framework. Left-hemispheric prefrontal excitation from anodal tDCS renders verbal markedness relatively more dominant, but this effect is not absolute. We discuss task contagion, study design, and individual differences in performance measures or tDCS response as possible contributors to systematic variation of the weights of multiple coding parameters for spatial-numerical associations.

## Introduction

Spatial associations can accompany seemingly abstract verbal concepts in highly intuitive ways. For instance, most individuals (in Western societies) tend to arrange their calendar schedules in left-to-right and top-to-bottom manner, and they tend to arrange numbers on physical layouts (such as computer keyboards) in a certain spatial direction (e.g., a left-to-right number line). Since the spatial dimension is immediately available in the human experience as the playground for physical action, it appears plausible that also verbal and symbolic-cognitive processes can mentally project onto space. Even more theoretically, it had been argued that sensorimotor interactions with the environment shape the understanding of increasingly abstract concepts such as sequential order or numerical magnitude in various theories of embodied cognition (Barsalou, 1999; Santiago et al., 2011; Fischer, 2012), child development and space-number acquisition (Patro et al., 2016), theories of magnitude (Walsh, 2003; Bueti and Walsh, 2009), or grounded cognition of serial order in working memory (Hurlstone et al., 2014; Abrahamse et al., 2017). The empirical behavior in experimental studies showcases the fascinating capacity of human agents to simulate and involve the spatial dimension also for concepts that are not directly physically available. But how do spatial associations emerge within the neurocognitive processing loop?

### Theoretical background

A good proxy measure for spatial associations of symbolic information is available in the Spatial-Numerical Association of Response Codes (SNARC) effect, which is evident in cognitive performance during very simple two-choice reaction tasks. When healthy participants classify features of sequential or numerical stimuli by key presses on the left- or right-hand side, the central finding of the SNARC effect consists in relatively faster left-hand over right-hand responses for small over large magnitudes (and initial over posterior sequence positions), and vice versa (Dehaene et al., 1993; Gevers et al., 2003; Wood et al., 2008; van Dijck and Fias, 2011). Regarding the neurocognitive processes beyond the SNARC effect, several theoretical positions are currently available in the literature and the exact mechanisms may be more multifaceted than initially declared, including the context of mental number representations in long-term memory (Hubbard et al., 2005), polarity correspondence (Proctor and Cho, 2006), working memory and / or spatial attention (van Dijck and Fias, 2011; Ginsburg et al., 2014; Abrahamse et al., 2016).

Regarding the underlying functional neuroanatomy, studies in numerical cognition have increasingly acknowledged the role of prefrontal-parietal circuits in intracortical recordings (Nieder and Dehaene, 2009; Nieder, 2016) or diffusion-tensor-imaging of white matter connectivity in human cortex (Klein et al., 2016). Thus, prefrontal regions appear to complement the established role of parietal regions in number representation (Dehaene et al., 2003) and spatial-numerical associations (Cutini et al., 2014). These recent results dovetail with theoretical accounts of the SNARC effects that predict prefrontal involvement in the form of verbal working memory. Using subthreshold neuromodulation of brain activity in prefrontal areas concurrent to respective tasks, studies with transcranial direct current stimulation (tDCS) can investigate and causally bolster these supposed linkages between prefrontal networks and subtle activation of implicit spatial associations of numbers and non-numerical sequences.

### Previous evidence

By testing the effect of left-hemispheric prefrontal tDCS on SNARC effects, we recently observed that inhibitory cathodal tDCS specifically blocked the generation of spatial associations in case of numerical symbols, but the stimulation did not affect performance when visuospatial distraction was directly available in the spatial displacement of stimuli (Schroeder et al., 2016). Precisely, in three sham-controlled cross-over experiments with either 1 mA anodal or cathodal tDCS, healthy participants were asked to classify centrally presented numbers. The task included left-hand or right-hand classifications according to features that were irrelevant to the spatial dimension, namely: number parity or magnitude. During a sham tDCS condition (which elicits comparable sensations, but no changes in brain activity), we obtained relatively faster response times for the congruent combinations of small (large) numbers and left-side (right-side) responding than for the incongruent combinations (e.g., small number and right-side responding), but this SNARC effect for single digits (1–9, without 5) was specifically abolished during another session of concurrent 1 mA cathodal tDCS (target cathode: left PFC, return anode: extracephalic placement on right upper arm). The effect of cathodal tDCS on SNARC effects was reproduced in two different paradigms (parity judgment and magnitude judgment, (Schroeder et al., 2016), experiments 1 and 2), but there were no general performance modulations in a control task, and polarity-specificity could be substantiated by a descriptive increase in SNARC effects during a separate experiment with excitatory anodal tDCS (Schroeder et al., 2016, experiment 3).

In a follow-up study, we investigated whether stimulation effects were specific for numbers or whether the polarity-specific tDCS effects would generalize to non-numerical sequence items. Having established a valid sham-control for modulations of spatial associations of numbers in the first study, we now tested different SNARC effects in a more economic parallel design with two groups of participants who performed baseline assessments (without tDCS) immediately followed by 1 mA anodal or cathodal tDCS applied concurrent to a second assessment of task performance (Schroeder et al., 2017b). The cardinal number sequence included number symbols 1, 2, 4, and 5, and the ordinal non-numerical sequence included written weekday names Monday, Tuesday, Thursday, and Friday (in German). In separate tasks, participants classified whether a number was smaller/greater than 3, and whether a weekday name came before/after Wednesday, by means of a left-side or right-side button press in congruent and incongruent blocks of trials. In line with the literature (Gevers et al., 2004), we observed left-to-right oriented SNARC effects during the baseline condition in the order-relevant magnitude judgment tasks for both sets of stimuli, with some variations across participant groups. The tDCS results, however, showed polarity-dependent dissociations of the directional arrangement (spatial associations) of the numerical vs. non-numerical stimuli. Specifically, in this previous study, participants displayed the reverse orientation for a SNARC effect for weekday stimuli (that is, Monday and Tuesday were faster responded to with their right hands, while Thursday and Friday were responded faster to with their left hands) when stimulated with anodal tDCS, but not with cathodal tDCS. For the group stimulated with anodal tDCS, we thus observed a striking reversal of the spatial association of a non-numerical sequence in the same two-choice reaction time testing paradigm, highlighting a dissociation between numerical and non-numerical sequential (weekday) spatial associations (Schroeder et al., 2017b).

However, weekdays are only one instance of non-numerical sequential stimuli. To conclude that the diverging stimulation results hold for non-numerical sequential stimuli in general, and not only specifically for weekdays, a conceptual replication with at least one other non-numerical sequence was required.

#### A multiple coding framework of SNARC-like effects

Theoretically, the obtained dissociation between SNARC effects for numbers and sequential stimuli (weekdays) was highly relevant and inconsistent with the view that spatial associations might be based on sequential order in general (van Dijck and Fias, 2011; Abrahamse et al., 2016). In a multiple-coding framework, we thus suggested that different mechanisms may all provoke spatial associations. We predicted at least three such codes:

- Spatial organization of numbers on a directed spatial mental number line,
- Sequential organization of items in verbal working memory,
- Markedness congruency.

Disruption or alteration of one dominant code by tDCS may lead to a greater influence of other codes contributing to the SNARC effect. Dependent on task, stimuli, and culture, these codes may all facilitate space-number or space-sequence associations in the same direction or sometimes also in opposite directions.

The account that multiple spatial associations may exist was recently put forward for codes (i) and (ii) in a study that showed spatial associations for both the numerical value of a number, but also its sequential position in a randomized working memory sequence (Huber et al., 2016). Earlier suggestions of different possible routes based on Arabic digits vs. language system were inspired by studies in German deaf signers' spatial-numerical and parity-space associations [e.g., Iversen et al., 2006, **Figure 3**, corresponding to codes (i) and (iii)]. Interestingly, the dominant activation of either routes was suggested to depend on stimulus attributes, e.g., lexical access modes for printed number words or sign language symbols for deaf signers.

As Abrahamse et al. (2016, p. 6) argued, the result of co-existing space-number and space-sequence associations (Huber et al., 2016) could be also explained by influx from long-term memory for heavily overlearned number magnitude associations from the Western participants' previous life experience. Such magnitude associations may have been pronounced in the Huber study particularly because a within-subject manipulation of number range could have drawn attention to number magnitude information. However, this same mechanism hardly accounts for reversed spatial associations of weekdays during anodal tDCS (Schroeder et al., 2017b), because retrieval of weekday spatial associations from long-term memory should also yield a left-to-right oriented SNARC effect in Western participants, but not a reversed one.

The diverging results from tDCS experiments allow to pinpoint theoretical claims in this regard: For example, if tasks were based on the same type of cognitive mechanism, stimulation should affect behavioral results in the same way. More specifically, activity-enhancing anodal PFC stimulation (most likely augmenting working memory; Ruf et al., 2017) should lead to an increase of sequential ordering of information. Vice versa, a reduction should only be observed if the influence of working memory is decreased by cathodal stimulation. Finally, definitely no stimulation should lead to a reversal of the SNARC effect. There is just no concept in the working memory account that predicts a reversal within the same people in a Western culture. In our view, one must assume an additional code responsible for such reversals to account for such data.

Because the third code in our model has seen less attention so far, we elaborate this one in a little bit more detail. The predictions of markedness correspondence specify the emergence of a reversed SNARC effect for *right-before* and *left-after* associations in the weekday task based on the linguistic markedness property of feature polarities (Berch et al., 1999; Iversen et al., 2004, 2006; Nuerk et al., 2004; see also: Proctor and Cho, 2006; Lakens, 2012, for conceptually similar theoretical accounts). Generally, this proposed mechanism draws on structural asymmetries in orthogonal verbal concepts such as utilized in SNARC tasks. For example, the *large* polarity can be considered the default endpoint of the magnitude dimension, whereas *small* constitutes its marked opposite category. The definition of marked vs. unmarked polarities can be reflected in default language usage, based on formal marking by a pre- or suffix (e.g., *male* vs. *female, efficient* vs. *inefficient*), distributional marking by usage restriction (e.g., *large* vs. *small;* large being more frequent), or specificity in semantic marking (e.g., *dog* vs. *bitch*, because the dog category can include both male and female dogs; therefor dog being unmarked). It should be noted, however, that the linguistic markedness concept has its theoretical barriers as well within linguistics (Haspelmath, 2006) and markedness-based effects on cognitive processing may be also found in non-linguistic stimuli as well (Proctor and Cho, 2006). Psychologically, the mere presence of marked features alone can lead to longer responses (e.g., Clark, 1969), as exemplified by lengthened responses to *odd* as compared to *even* (Hines, 1990).

The concepts of structural symmetry and polarity correspondence moreover posit that response selection in classification tasks should be faster when the markedness or polarities of stimulus and response alternatives are matched, which was found to be true for a series of effects (Proctor and Cho, 2006; Proctor and Xiong, 2015). One remarkable example for such correspondence effects is the association between parity status and spatial responding in a direction consistent with the linguistic marking of *left* and *odd* feature polarities (Berch et al., 1999; Nuerk et al., 2004), at least for right-handers (Huber et al., 2014). Based on this concept, interestingly, also the regular *left-small* and *right-large* associations between space and numerical magnitude in the default SNARC effect for numbers can be explained (e.g., Schroeder and Pfister, 2015), because *small* and *left* are considered to be marked. Yet, it is important that the markedness code alone cannot explain all SNARC results. Another coding mechanism must be dominant for left-to-right oriented SNARC effects of non-numerical sequences in order classification tasks, because *after* and *left* polarities are considered to be marked and their combinations should facilitate responding (as in Bächtold et al., 1998, experiment 2, with before-after judgments of numerical stimuli), which is, however, not the case under usual circumstances and without concurrent tDCS (Gevers et al., 2003, 2004; Schroeder et al., 2017b, in press).

The suggestion of an activated dominant route among several possible cognitive processing alternatives can be substantiated by additional previous results. For example, space-number associations were found to be resilient to modulations of spatial response polarity by keyboard eccentricity (Santiago and Lakens, 2015). Another interesting example was found in selective number magnitude associations with “left” vs. “right” vocal responding, and parity associations with “good” vs. “bad” vocal responding, but not vice versa, which constrained the validity of a general polarity account [e.g., exclusively (iii); Leth-Steensen and Citta, 2016; see also Fischer et al., 2016, for vocal space-number associations in children]. Further advancing this recent result of selective associations, another independent tDCS study showed reduced parity-space (but not magnitude-space) modulations from 1.5 mA cathodal tDCS over the parietal cortex (returned at supraorbital location; Di Rosa et al., 2017). While polarizing entirely different networks than the prefrontal-extracephalic configuration used in our studies, their dissociation results from tDCS converge to the notion that numerical SNARC effects may preserve based on alternative codes [such as (i) or (ii)] during neuromodulation, whereas other effects that depend much more on markedness-based processing [e.g., parity-space associations, (iii)] would deteriorate.

Apparently, flexible switching between spatial association mechanisms by the active anodal tDCS condition in our previous study (Schroeder et al., 2017b) would have produced the observed dissociation by rendering the markedness process (iii) most dominant during left-hemispheric prefrontal excitation by anodal tDCS. The presented multiple codes framework is also consistent with previous dissociations of spatial associations for numerical and non-numerical sequences in some observations of hemispheric neglect (Zorzi et al., 2006; Zamarian et al., 2007), that showed different (reversed) error patterns for bisections of non-numerical as opposed to numerical sequences. Moreover, the multiple-coding account dovetails with orthogonally oriented spatial associations for auditory and verbal presentations of month names in a case of sequence-space synaesthesia (Jarick et al., 2009). In sum, we believe the multiple code framework can account for a multitude of SNARC-like effects with and without tDCS.

### Aim of the study and hypotheses

In contrast to the stable and replicated effect of cathodal tDCS (Schroeder et al., 2016), the overall effect of anodal tDCS was less clear. For instance, although the reversal of spatial associations of weekday stimuli during anodal tDCS was theoretically meaningful and statistically clear-cut in our previous analysis (Schroeder et al., 2017b), results were based on a single tDCS study in parallel design without sham control condition. Thus, in the current study, we set out to challenge the robustness of our previous finding and to test the generalizability to another set of sequential stimuli. Moreover, by testing month stimuli as another non-numerical sequence, we also tested the presented multiple-coding framework which would predict dissociations between number and month stimuli by anodal tDCS, unlike potential unifying accounts.

The full publication of such replication attempts is particularly important in the domain of tDCS to transparently address the contemporary skepticism among researchers and practitioners. Specifically, the conception of the neuromodulation technology is rather reserved and some recent articles have sparked doubt on its potential effects in quantitative review (Horvath et al., 2015) or have outlined large variability in physiological measures of motor cortex excitability modulations (Strube et al., 2016). Due to publication biases, it is possible that an even larger number of negative results had not been reported on, but underlying reasons remain elusive. For instance, we found in our previous results that the efficacy of modulation depended on the level of cognitive activity induced by a task (Zwissler et al., 2014) or on the timing of stimulation in a first, second, or third session of repeated task performance (Dockery et al., 2009). Moreover, if a task classification rule incorporated a stimulus dimension not necessarily recruiting the respective (prefrontal) network (Fias et al., 2001), stimulation was not effective (Schroeder et al., 2017b). These findings line up with the general theoretical premise of state dependency of transcranial brain stimulation (Silvanto and Pascual-Leone, 2008; Fertonani and Miniussi, 2016).

The aim of the current study was therefore threefold. First, following the striking reversal of the weekday sequence spatial associations, we sought to replicate the reversal of a non-numerical ordinal sequence with anodal tDCS in a sham-controlled design. Our primary outcome was defined as differences in the unstandardized regression coefficients capturing the SNARC effects (Fias et al., 1996; see Data Treatment in the next section) between sham and anodal tDCS condition, which should resemble the observed difference between baseline and anodal tDCS in the same parameter in our previous study (Schroeder et al., 2017b, p. 43, paired *t*-test on unstandardized regression coefficients). We decided to change the experimental design in order to establish a valid sham-control that elicits comparable sensations and thus to control for any motivation-related changes (although it has to be noted that we could not observe significantly different sensations between anodal and cathodal tDCS before). In the present study, participants were tested on separate days with a minimum wash-out period of 48 h to circumvent any possible long-term neuroplastic (after-) effects of the stimulation. Second, to explore whether the observed directionality switching was directed by the verbal presentation format of weekday stimuli (which is somewhat related to the theoretical markedness correspondence account), we now also presented single-digits in their verbal, i.e., written, form (i.e., German word “eins” for “1,” and so on). Finally, we included another non-numerical sequence task with month names to examine the generalizability of our original results. With the same month names used in the new task (albeit in Dutch language), previous experiments demonstrated a spatial mapping from left-to-right akin to the SNARC effect for numbers (Gevers et al., 2003). Thus, the third experiment on month names was specifically designed to conceptually replicate our finding with another non-numerical sequence and to test the predictions of the presented multiple-coding account. Previously, we had suggested that the dissociative tDCS effect in weekdays was driven by the markedness features of their ordinal item structure (i.e., as weekdays constitute a non-numerical sequence). If these conclusions hold, similar effects should be obtained with other non-numerical sequences as well, i.e., also with month names. We hypothesized that 1 mA anodal tDCS would reverse the spatial associations of both weekdays and month names, but that stimulation would rather yield a small enhancement in opposite direction of regular left-to-right SNARC effects for numbers.

## Methods

### Participants

Based on a-priori power calculation (see respective section on the next page), healthy volunteers (*N* = 24, mean age: 21.8 year, range: 18–26 year, 5 males) were recruited from the general and student population. All participants attended a sham and a stimulation session with 1 mA anodal tDCS, in counterbalanced order (mean inter-session interval: 5.1 days, range: 2–9 days). This study was carried out in accordance with the recommendations of University Hospital Tuebingen Ethical Commision with written informed consent from all subjects. All subjects gave written informed consent in accordance with the Declaration of Helsinki. The protocol was approved by the University Hospital Tuebingen Ethical Commision (Number of approval: 701/2015BO2). Eligibility for the study was assessed by self-reports collected prior to the first experimental session. Right-handed volunteers (according to the questionnaire by Oldfield, 1971) were eligible for participation if they were native German speakers without neurological or psychiatric impairments and if they fulfilled tDCS safety requirements (no metallic implants, cardiac pacemaker, pregnancy, medication, or use of recreational drugs). Participation was compensated with money or student credits. The experiments were performed in Tübingen between June-November 2016.

### Anodal transcranial direct current stimulation (tDCS)

The study followed a sham-controlled cross-over design. Transcranial direct current stimulation (tDCS) was administered in either active anodal or sham configuration on separate testing days in counterbalanced order across all participants. Direct current was generated by a CE-certified stimulator (neuroConn GmbH, Illmenau, Germany). Sham stimulation was realized by fading out the direct current after only 40 s of stimulation, yet participants started the first task in both conditions always after 5 min following the tDCS fade-in. In contrast, active anodal tDCS was administered continuously at 1 mA intensity for a total duration of 25 min (“online” to the task). In contrast to offline tDCS effects (i.e., behavioral effects are assessed after the termination of stimulation), online tDCS effects can be linked directly to resting membrane threshold changes in cortical excitability as opposed to longer-lasting neuroplastic responses. A minimum wash-out period of 48 h was imposed between sessions. The target anode (5 × 7 cm) was fixed over F3, the return cathode (5 × 7 cm) was fixed over the right upper arm. Impedances were below 10 kΩ. Participants rated adverse effects (Brunoni et al., 2011) after completion of the stimulation protocol.

This specific anodal tDCS protocol was motivated by our previous observation that the reversal of SNARC effects for non-numerical ordinal words was induced by 1 mA anodal, but not by 1 mA cathodal tDCS over the left PFC with extracephalic return electrode. In contrast to the previous study, the cross-over study design was implemented here to establish a valid sham control.

### Tasks and stimuli

Participants completed all three experimental tasks during both sessions either following a 4:20 min stimulation-free rest phase in the sham session or concurrent to the anodal tDCS following 5 min of at-rest stimulation. All tasks were completed online in the active anodal tDCS session.

Since we observed significant modulations of spatial associations exclusively during the most active tasks with explicit comparison instructions (Schroeder et al., 2017b), the testing sessions in this study included only the order-relevant magnitude comparisons in all three stimulus set variants.

The tasks closely followed previous order-relevant implementations (Gevers et al., 2003, 2004). In all three tasks, participants had to repeatedly classify stimuli by following a response-mapping rule and by operating two keyboard buttons with their right-hand and left-hand index fingers (covert “s” and “l” on a QWERTZ-keyboard). Stimuli included a numerical sequence in German verbal notation (“eins,” “zwei,” “vier,” “fünf”; English translation: “one,” “two,” “four,” “five,” respectively), the weekday sequence (“Montag,” “Dienstag,” “Donnerstag,” “Freitag”; English translation: “Monday,” “Tuesday,” “Thursday,” “Friday,” respectively), and the month series (“Januar,” “Februar,” “März,” “April,” “September,” “Oktober,” “November,” “Dezember”; English translation: “January,” “February,” “March,” “April,” “September,” “October,” “November,” “December,” respectively). The task instruction was to classify whether a number was smaller or larger than “drei” (three), whether a weekday was before or after “Mittwoch” (Wednesday), and whether a month was before or after “Juli” (July).

Response assignments to the classification decisions (e.g., right = large or right = before) were alternated within the tasks in counterbalanced order across participants. Thus, participants always solved two blocks with “compatible” and “incompatible” response assignments (e.g., a left-hand assignment to small numbers/before positions would be considered to be compatible following the standard results of relatively faster responses in this block as compared to the opposite right-hand assignment).

**Figure 1:**
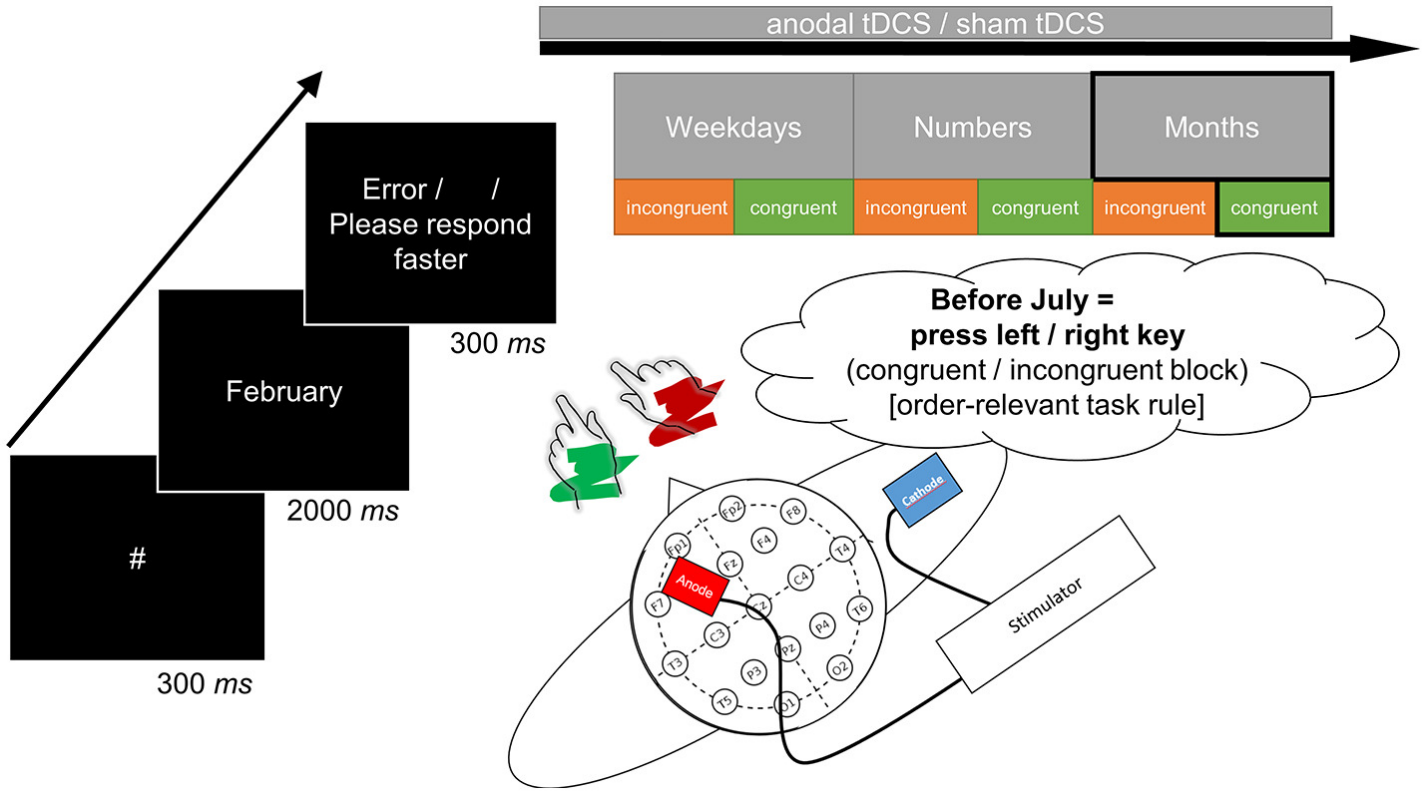
The structure of a single trial as exemplified by judgment of the month name “February” (left-side). The correct response in the respective incongruent or congruent task block was determined by the order-relevant task rule based on sequential position in the month sequence (before/after). In this case, the congruent block would afford a left-hand response for classification of “before” whereas the incongruent block would afford a right-hand response for classification of “before.” Congruent and incongruent blocks (in counterbalanced order across participants) were presented sequentially for all three sets of stimuli (upper-side). The target anode was placed over F3 and the return cathode over the right upper arm to avoid polarization of another brain area (lower-side). All tasks were initiated and terminated concurrent to anodal tDCS in the verum session (online stimulation).

All single tasks were preceded by a brief training block (16 trials) and an error count emphasized correct responding. The experimental procedure comprised 40 target repetitions in each task, with 300 ms fixation hash (#), 2 s target presentation (or until response), and conditional 300 ms error feedback (German words “Fehler” [error] or “Bitte schneller antworten” [please respond faster]) or a blank inter-trial interval. Figure 1 displays a schematic of a trial. Note that consequently, because the month task included twice as many stimuli as both other tasks, experimental blocks took longer for this task. There was the possibility to take self-paced breaks between tasks and blocks.

The order of the three experimental tasks (number words, weekdays, months) was balanced across participants in a Latin Square Design, resulting in three conditions (WMN, MNW, NWM). Half of all participants started each task with the incongruent response mapping.

### A priori power calculation

A power calculation was performed to estimate sample size based on the previously observed reversal effect (Schroeder et al., 2017b) using the program MorePower 601 (Campbell and Thompson, 2012). In our previous experiment, the observed effect size of anodal tDCS on weekday stimuli in the within-group comparison was *d* = 0.95. Assuming this effect size, the sample size for a replication (at α = 0.05 and a priori power of 1–ß = 0.99) was calculated to require *N* = 24 participants (dependent samples *t*-test of unstandardized regression coefficients).

### Data treatment and statistical analysis

Only correct trials were considered for analyses of response times (RTs, 95.4%). Additional post-error trials (3.6%, to reject systematic RT variation in post-error slowing; Notebaert et al., 2009) as well as stimulus-repetition trials (17.9%) were rejected from analyses (SNARC effects are reduced in stimulus repetition trials due to episodic memory; Tan and Dixon, 2011; Pfister et al., 2013). Next, response latencies that deviated more than 3.0 SD from the respective cell mean (computed separately for each target-response combination) were rejected (1.0%). These criteria left 73.0% of all trials available for subsequent analysis. The rationale of this data treatment was to reject trials that included systematic RT variation due to other known cognitive effects. Following a reviewer's comment, and acknowledging the fact that different analysis strategies could in principle yield different outcomes (Silberzahn et al., 2017), we also demonstrate in the Supplementary Materials (SA1) that the main results did not change substantially in an alternative analysis based on correct mean RT without further trial rejection.

**Figure 2:**
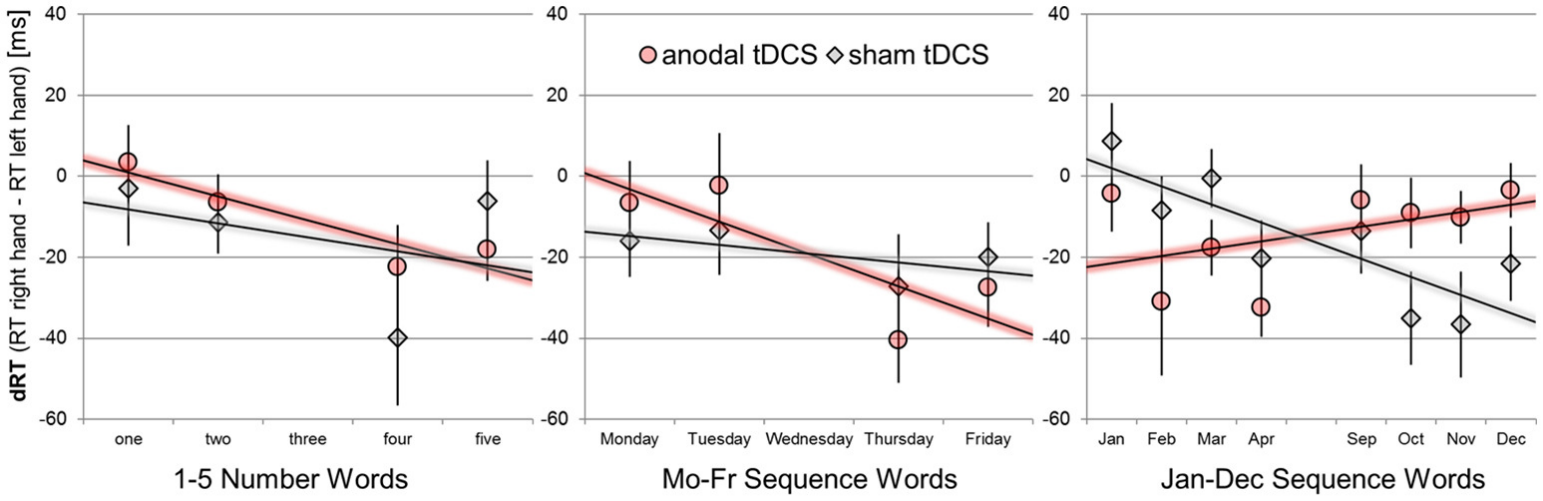
Mean response hand differences (dRT = RT right-hand–RT left-hand) as a function of sequence position/numerical magnitude for the number stimuli, weekday stimuli, and month names. Performance during sham tDCS is indicated by gray diamonds, performance during anodal tDCS by red circles. Error bars indicate standard errors of the mean. Negative-signed linear regression lines indicate regular SNARC-like spatial associations. Note that only four predictor variables for magnitude/order position were used in all statistical analyses and thus consecutive month names were aggregated (e.g., Jan+Feb = position 1), unlike shown in the graph.

We used unstandardized regression coefficient analyses^1^ to extract the relative advantage of right-hand over left-hand responses with increasing numerical magnitude or sequence position, as it is usually implemented to quantify SNARC effects (Lorch and Myers, 1990; Fias et al., 1996) for numerical and non-numerical sequence stimuli (Gevers et al., 2003, 2004). The computation involves several steps: First, RT right-hand—left-hand differences are determined separately for each participant, stimulation condition, task set, and target stimulus combination from median RTs. Next, RT hand differences are predicted by numerical magnitude (1-5) or sequence position bins (numerically coded as 1-5; see Figure 2). A negative regression coefficient (tested with one sample *t*-tests against zero) indicates the typical behavioral pattern of SNARC effects, that is: Participants give relatively faster left-hand than right-hand responses to small/before stimuli and participants give relatively faster right-hand than left-hand responses to large/after stimuli. The resulting regression coefficients were submitted to separate paired-samples *t*-tests for each task (number, weekdays, months) to outline the effect of anodal tDCS.

## Results

### Adverse sensations of tDCS

Adverse effect ratings for both sham and active anodal tDCS are summarized in Table 1. Participants reported slightly more “tingling elsewhere” [*t*_(23)_ = 2.01, *p* = 0.057], but all values were relatively close to the lower boundary of the self-report scale (see Table 1).

**Table 1:** TDCS adverse effects. Adverse sensations were assessed on a 5-point Likert-like scale after each session (1 = none, 5 = extensive).

**Adverse Sensation**	**Anodal tDCS *M (SD)***	**Sham tDCS *M (SD)***	***p***
Tingling at the site of the electrode	2.00 (0.78)	2.17 (0.87)	0.26
Tingling elsewhere	1.29 (0.55)	1.08 (0.28)	0.06
Exhaustion	1.61 (1.03)	1.87 (0.97)	0.31
Itching	1.65 (0.64)	1.65 (0.88)	0.99
Headache	1.22 (0.67)	1.26 (0.62)	0.81
Nausea	1.00 (–)	1.00 (–)	–

*Ratings following anodal tDCS and sham tDCS were subjected to paired t-tests*.

### Stimulation effects

#### One-to-five number words

SNARC effects with negative-signed coefficients were significantly different from zero for the performance during anodal tDCS [*b* = −6.74 ms/bin; *t*_(23)_ = −2.28, *p* = 0.032], but not during the sham tDCS condition [*b* = −4.16 ms/bin; *t*_(23)_ = −0.88, *p* = 0.386]. The difference in SNARC effects was not significant [*t*_(23)_ = −0.53, *p* = 0.533].

#### Monday-to-friday sequence words

SNARC effects with negative-signed coefficients tended to be different from zero for the performance during anodal tDCS (*b* = −8.75 ms/bin; *t*_(23)_ = −1.99, *p* = 0.057], but not during the sham tDCS condition [*b* = −3.09 ms/bin; *t*_(23)_ = −0.75, *p* = 0.462]. The difference in SNARC effects was not significant [*t*_(23)_ = −0.75, *p* = 0.460].

#### January-to-december sequence words

A reversed/abolished SNARC effect with positive-signed coefficient was not significant during the anodal tDCS condition [*b* = +4.22 ms/bin; *t*_(23)_ = 1.40, *p* = 0.175]. The SNARC effect for the same month stimuli was negative-signed during the sham condition [*b* = −7.45 ms/bin; *t*_(23)_ = −2.00, *p* = 0.057]. In direct comparison of the two stimulation conditions, the effect of anodal tDCS was significant [*t*_(23)_ = 2.68, *p* = 0.013, *d* = 0.55].

#### *Post-hoc* power analysis

Overall, the results of the current study corroborate our hypotheses. With number words as target stimuli in a magnitude comparison task, we observed a descriptive increase in the regular left-to-right SNARC effect with anodal tDCS, but, in line with our previous studies, this presumable modulation effect was relatively small and not substantiated by statistically significant differences (Schroeder et al., 2016, 2017b). With weekday stimuli, we could not detect the previously observed reversal of spatial associations in the sham-controlled design, and the direction of a possible effect was descriptively reversed. With month stimuli, we observed a significant effect of anodal tDCS, conceptually replicating the recent result of anodal tDCS on non-numerical spatial associations (Schroeder et al., 2017b). However, the effect size of this modulation was reduced to approximately half of the effect size in the original observation (within-subject test of reversal relative to baseline performance: *d* = 0.95). For the paired samples *t*-test and the effect size observed here (*d* = 0.55), the current sample size of *N* = 24 resulted in a *post-hoc* power of 1–ß = 0.73.

#### Replication of dissociation between numerical and non-numerical sequence

An important observation of our previous study (Schroeder et al., 2017b) was the dissociation between the numerical and non-numerical sequence by anodal tDCS. This observation is in conflict with the proposal of a unified WM account of the SNARC effect (van Dijck and Fias, 2011; Abrahamse et al., 2016). Thus, we particularly tested the dissociation between the spatial associations of number and month sequences by anodal tDCS in the current data. The resulting clear interaction [*F*_(1, 23)_ = 9.19, *p* = 0.006, $\eta_{\text{p}}^{2}$ = 0.29] was in line with our previous observation (Schroeder et al., 2017b). In more detail, the interaction effect was substantiated by a significant difference in SNARC effects for number and month stimuli during anodal tDCS [*F*_(1, 23)_ = 5.81, *p* = 0.024, $\eta_{\text{p}}^{2}$ = 0.20], due to a negative coefficient for numbers and a positive one for months (see above), but the difference was not significant during sham tDCS [*F*_(1, 23)_ = 0.50, *p* = 0.486].

### Joining data from previous studies

To address the shortcoming of reduced power due to smaller effects than anticipated, we decided to resubmit the previously obtained regression coefficients from earlier data sets to joint analyses. The respective experiments and their main findings are fully reported in Schroeder et al. (2016, experiment 3; S16) and Schroeder et al. (2017b; S17). Here, we re-analyzed only those datasets which were collected during the exact same anodal tDCS configuration and the respective sham or baseline control conditions.

#### Effect of anodal tDCS on spatial associations of number stimuli

Data from three experiments yielded a total *N* = 72 (drawn from the current study, from S16, and S17). Regression coefficient analyses were run as described above and pseudo magnitude-bins (1, 2, 4, and 5) were assigned for the larger number range (1–9, S16). Coefficients were submitted to a mixed ANOVA with the repeated-measures factor *tDCS* (anodal vs. sham / baseline stimulation) and with the between-subjects factor *experiment* [1–5 (S17), eins-fünf (current study), 1-9 (S16)].

**Figure 3:**
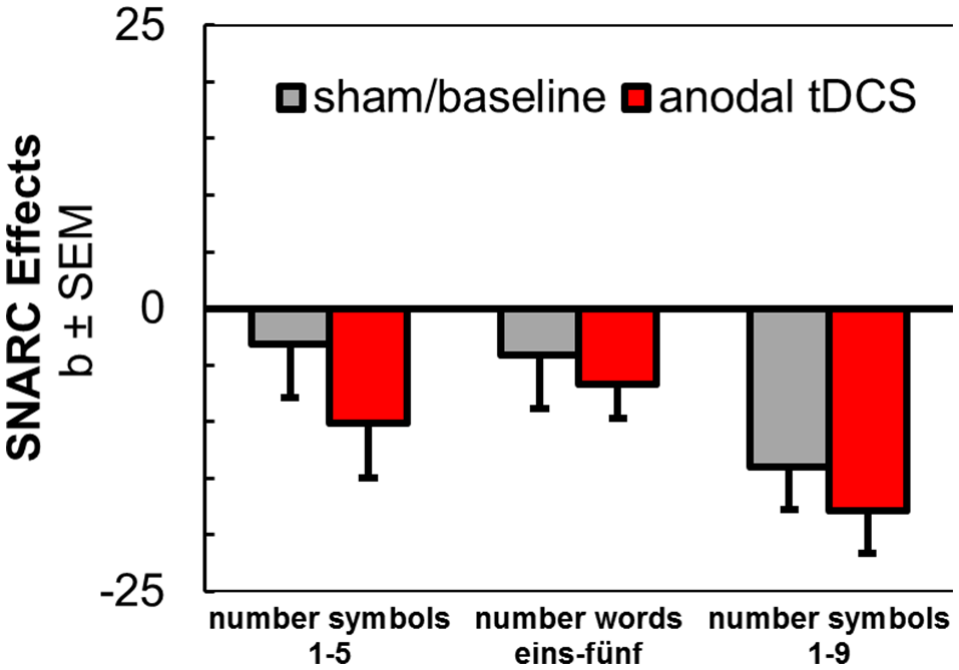
Aggregated results from anodal tDCS during SNARC tasks with number symbols 1–5 (Schroeder et al., 2017b), number words (current study), and number symbols 1–9 (Schroeder et al., 2016).

The main effect of *tDCS* was barely significant in this analysis [*F*_(1, 69)_ = 2.82, *p* = 0.098, $\eta_{\text{p}}^{2}$ = 0.04]. There was no interaction with the between-subjects factor *experiment* [*F*_(1, 67)_ = 0.24, *p* = 0.784], and SNARC coefficients tended to be larger for the extended number range (1–9) in S16, possibly due to the assignment to pseudo magnitude-bins when extracting coefficients, due to individual differences, or due to the nature of the parity judgment task [see Figure 3; *F*_(1, 69)_ = 2.66, *p* = 0.077, $\eta_{\text{p}}^{2}$ = 0.07]. Interestingly, this pattern also resembled the steeper SNARC slopes in the present experiment for the month series during sham tDCS, comprising an extended range of stimuli as compared to the employed weekday and number series.

For a directed paired-samples *t*-test (one-tailed) with data from all participants of the three studies, the effect of anodal tDCS was significant [*t*_(72)_ = 1.70, *p* = 0.047], but the standardized effect size estimate was small (*d* = 0.21). Even for this least conservative test, the post-hoc power to detect the stimulation effect in the aggregated sample was insufficient (1–ß = 0.53).

#### Effect of anodal tDCS on the spatial associations of non-numerical sequences

Data from three experiments (drawn from the current two experiments and from Schroeder et al., 2017b) yielded a total *N* = 72^2^. The main effect of *tDCS* was significant [*F*_(1, 69)_ = 7.18, *p* = 0.009, $\eta_{\text{p}}^{2}$ = 0.09]. However, the stimulation effect was further qualified by a significant two-way interaction with the between-subjects factor *experiment*, which statistically confirmed the different results for weekday stimuli in the two studies [*F*_(2, 69)_ = 5.33, *p* = 0.007, $\eta_{\text{p}}^{2}$ = 0.13]. Independent samples *t*-tests showed that SNARC effects for weekday sequence words tended to be somewhat larger during sham/baseline tDCS in our previous study (*b* = −14.84 ms/bin) than in the current study [*b* = −3.09 ms/bin; *t*_(42.04)_ = 1.68, *p* = 0.101], but not larger than in the month sequence [*b* = −7.45 ms/bin; *t*_(39.74)_ = 1.09, *p* = 0.282]. The reversed SNARC effect during anodal tDCS in the previous study (*b* = +8.30 ms/bin) was significantly different from the result collected during stimulation in the current experiment [*b* = −8.77 ms/bin; *t*_(46)_ = 2.83, *p* = 0.007], but not different from the reversed/abolished SNARC effect for month stimuli during anodal tDCS [*b* = +4.22 ms/bin, *t*_(26)_ = 0.80, *p* = 0.429]. Thus, the results corroborate a potential effect of the most prevailing experimental differences between the two studies, i.e., the study design (parallel vs. cross-over design), the presence of different control tasks (color judgment tasks or testing of month stimuli with more sequential positions), but also the individual differences in the inclination of SNARC effects already during the sham / baseline session.

## Discussion

The purpose of the current experiments was to extend on the possibility of switching between spatial associations by concurrent administration of prefrontal anodal tDCS. Three series of stimuli were used in simple classification tasks that usually elicit spatial associations in the SNARC effects for number symbols (German words eins-fünf), weekdays (Montag-Freitag) and month names (Januar-Dezember). We observed a small but non-significant increase^3^ in spatial-numerical associations for number symbols from anodal tDCS. However, after including additional empirical data from our previous experiments (Schroeder et al., 2016, 2017b), we here also report for the first time that anodal tDCS can significantly enhance spatial-numerical associations (e.g., the regular SNARC effect) in left-to-right direction, but the effect is only evident in the least conservative test and with small effect size (*d* = 0.21). This systematic effect was always descriptively available in our earlier studies and a modulating effect of notation or number range appeared negligible in our analysis (see Figure 3).

**Figure 4:**
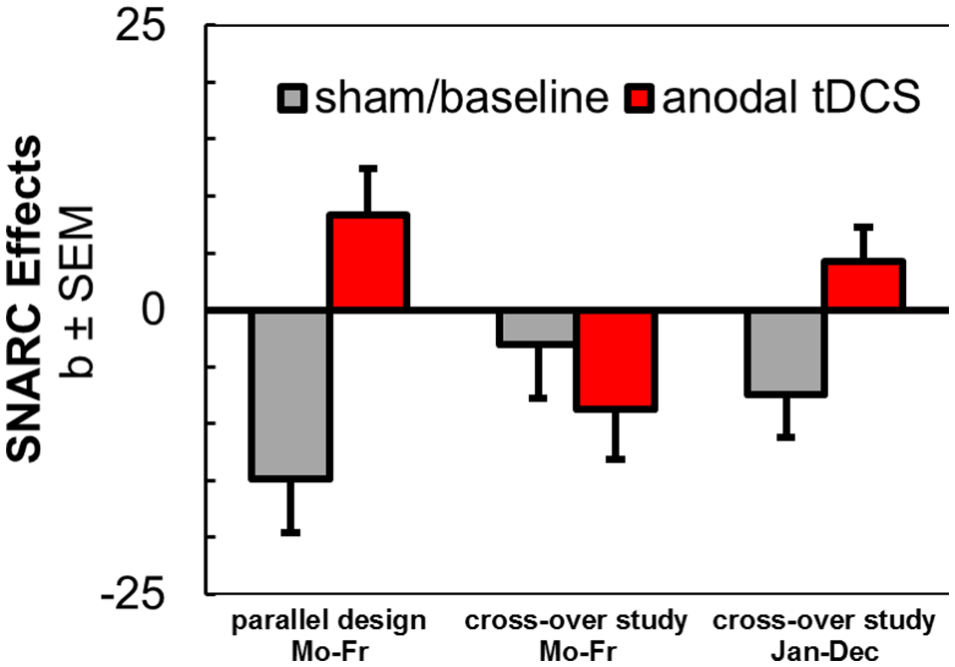
Aggregated results from anodal tDCS during SNARC tasks with weekday stimuli in parallel design (Schroeder et al., 2017b), in cross-over design (current study), and with month stimuli in cross-over design (current study).

Most importantly, however, we could partially replicate our previous observation of a reversal of spatial associations of a non-numerical sequence by anodal tDCS (Schroeder et al., 2017b). In line with our original hypotheses, tDCS successfully modulated spatial associations of a non-numerical sequence in another series of stimuli (month sequence). During anodal stimulation, participants produced relatively faster responses with their left hands to months in the second half of the year, but they showed relatively faster responses with their right hands to months in the first half of the year, which was opposed to their behavioral performance during sham tDCS. With these stimuli, we reproduced the tDCS dissociation between spatial associations of a numerical and a non-numerical sequence, further challenging potentially unifying theoretical accounts. However, the conceptual replication of the reversal of the weekday sequence by anodal tDCS was not successful in the sham-controlled cross-over design and descriptively even opposite to our previous result (see Figure 4).

It should be noted that the positively signed SNARC coefficient for month stimuli during anodal tDCS was not significantly different from zero. Thus, the coefficient test could not secure the implication that this spatial association was reversed and it remains possible that anodal tDCS instead abolished the SNARC effect in the current study.

Three reasons could potentially account for the diverging results of weekdays in this study as compared to the reversal effects for weekdays in our previous study and month in the current study: (1) The stimulation effect is not true (Type I error of two observed reversals), (2) the second experiment of our current study was underpowered (Type II error of current result), or (3) other systematic reasons rendered the tDCS procedure ineffective in the case of weekday stimuli in the current study. We discuss each of these issues separately.

### Type I error: false positives in previous results?

In recent years, it has come to public awareness that Type I error rates (false positives) are particularly pronounced in psychological research under the umbrella of reproducibility crisis (Open Science Collaboration, 2015), supported by publication biases, questionable research practices, and other factors that lead researchers to publish and report only on successful (“significant”) manipulations in their studies (Nosek et al., 2015). Relative to the impact of the current research topic, and also the controversy around tDCS effects on cognition, it seems reasonable to assume that some publications on the efficacy of tDCS could include Type I errors. False positives rates can inflate with inclusion of more measurement factors or tasks, which is particularly critical when only positive procedures are reported on.

Over the current set of studies, we believe that the pattern of results and especially the successful partial replication in the month sequence does not point toward a false positive in the original results (Schroeder et al., 2017b). Actually, in series of experiments, the probability that one replication would not work is statistically plausible and hinges on the power of the test. For a simplified example, given a power of 73% (i.e., the power to detect the effect of anodal tDCS on the month series in the current experiment), the chance for 3 out of 3 experiments to turn positive is relatively low (0.73^3^ = 38%). In contrast, the probability to obtain 2 out of 3 significant results (i.e., as it was the case here) would be even slightly higher (43%), although this example assumes the case that the effect is actually true (see Francis, 2012 for discussion of this too-good-to-be-true approach).

### Type II error: lack of power for conceptual replication in current results?

Following up on the observed power of 0.73 (related to the reversal of spatial associations for the month sequence) it could be plausible that a tDCS effect was simply not observed in the weekday sequence due to a Type II error. Actually, running low-powered studies has a long tradition in clinical psychology and estimates were below 50% chance to detect a medium-sized effect (i.e., *d* = 0.5 or larger), but power was acknowledged only in most seldom cases (Sedlmeier and Gigerenzer, 1989). Importantly, the failure to detect an effect in null hypothesis significance testing does not provide evidence for absence of the effect in the first place.

On this occasion, it is revealing to notice that effect sizes diverged tremendously between studies. A priori sample size estimation initially suggested sufficient power with the included sample size, but the post-hoc power analysis deviated from the a priori sample size estimation due to the diminished effect size. Although it is acknowledged that initial discoveries of psychological effects may report larger effect sizes than replications (with a standardized mean difference of 0.21, Open Science Collaboration, 2015, p. 5), the reduction in effect size in the present case may have been also supported by other systematic differences between studies.

### Systematic reasons for partial failure of replication

However, while power issues could in principle account for our results pattern, there are also reasonable alternative accounts suggesting that the obtained results are due to systematic underlying differences between our previous study and the current results. For example, the facts that other stimuli and control tasks were tested in different study designs (i.e., parallel vs. cross-over design) could play into the obtained results. Although all stimuli comprised sequence items, the month series includes a larger number of items than both number and weekday sequences, which could have influenced also the mapping of the relatively smaller item sets onto spatial templates. Actually, our joined analysis showed larger SNARC effects for extended number ranges, which dovetails with the differences in SNARC effects for different stimuli of the present study during the sham condition (showing the largest effect in the extended month range).

Next, since the markedness correspondence account attests different possible strategies to produce spatial associations from verbal markedness, but also from other mechanisms such as visuospatial simulation or serial-order processing, it may be possible that detachment from a previous task set (e.g., classification of month stimuli) to perform on a new, yet comparable task (e.g., classification of weekday stimuli) involved a subtle switch in the spatial association strategy used to allow for more effective task set representations. Thus, we speculate that the presence of different tasks in the same session could lead to contagion to another spatial association mechanism in changing item sets.

Furthermore, the possibility of individual differences could be relevant for both the modulation with tDCS and also for the effect inclined by our tasks. Regarding the SNARC effect, variation is remarkable in general and only ~70% of the general population present regular spatial-numerical associations (Wood et al., 2006; Cipora and Nuerk, 2013). We also noted that different inclinations of SNARC effects for weekdays during sham/baseline were present in the current study and the previous study, which could suggest a lack of modulation effects due to the lack of sham effects in the present sample (see Figure 4). Specifically, the SNARC effect in the sham tDCS condition trended toward being negative only in the month order classification task, but not in the numerical magnitude or weekday order classification tasks. Moreover, there is also noticeable variability in the responses to tDCS, already when motor cortex stimulations and physiological measurements are performed (Wiethoff et al., 2014; Strube et al., 2016), which could result in different effectivities as also captured by the effect size of the tDCS modulation in the month task.

In any case, all presented possibilities remain post-hoc hypotheses generated from the data and thus they need to be submitted to respective confirmatory testing in the future, since they also raise potential relevance to the systematic investigation of tDCS effects in general.

### Toward an extended multiple-coding framework of space-metric associations

**Figure 5:**
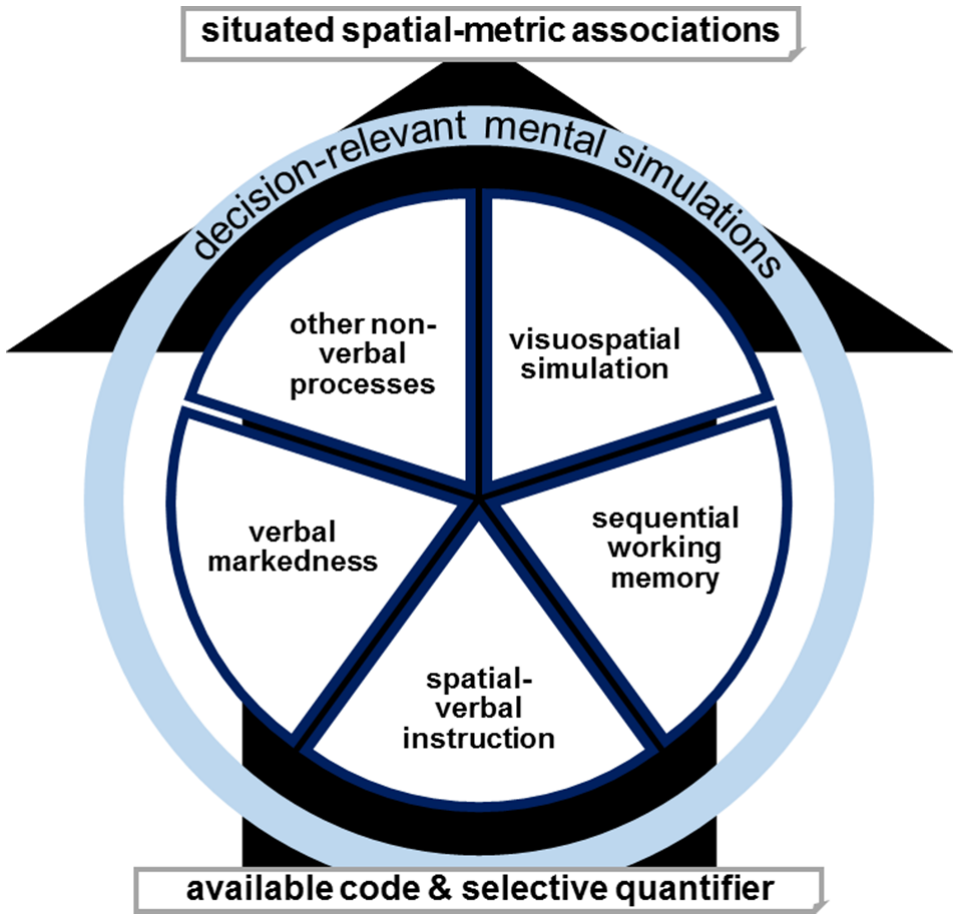
The availability and selectivity of a quantifier decides for the dominant involvement or combination of verbal markedness, spatial-verbal instruction, sequential working memory, visuospatial simulation, or other non-verbal processes in determining situated spatial-metric associations. Decision-relevant mental simulations in either form result in behavioral effects. Targeted tDCS (or another manipulation) can modulate either code and thereby also influence the dominance of the remaining simulations. For example, targeted activating tDCS augments a specific code or codes dependent on the topography of stimulation and the corresponding network.

We assume that spatial associations can result from different mechanisms and we propose several verbal and non-verbal simulation processes (see Figure 5). In this multiple-coding framework, anodal tDCS of left-hemispheric prefrontal circuits could facilitate the processing of verbal markedness, render correspondence effects between right-before and left-after classifications as task-relevant, and thus result in the observed reversal of SNARC effects for the ordinal sequence (Schroeder et al., 2017b). For the numerical sequence, the same mechanism would enhance correspondence effects between large-right and small-left classifications as task-relevant and thus result in the regular left-right direction of the SNARC effect (Schroeder and Pfister, 2015). However, in this theoretical model, it is possible that also other verbally mediated strategies (such as working memory mechanisms of sequential order) or visuospatial simulations produce spatial associations that are resistant to a certain stimulation, especially considering the possibilities of individual differences or task contagion. Such a multiple-coding framework also agrees with the inconsistent outcomes for non-numerical stimuli, as it was actually observed in the weekday stimuli in the present study.

For a conceptualization of the neurocognitive processes involved in mentally aligning (properties of) objects with physical space, the results collected here basically reiterate the connotations drawn from the previous dissociation of spatial associations of number and sequence (Schroeder et al., 2017b, in press). Further support for the different coding strategies comes also from a recent dissociation of parity-space and number-space associations during tDCS over left and right parietal cortices, where only the former were modulated by cathodal tDCS (Di Rosa et al., 2017). Complementary to these results, the current study underscores that the spatial alignments of both numerical and non-numerical sequences are guided by prefrontal activity, albeit with orthogonal responses to tDCS polarities. Furthermore, the data highlight a critical dissociation between numbers and months, which opposes a unified theoretical account and provokes a multiple-coding framework.

The systematic reasons outlined above can be easily arranged along the proposed model by emphasizing the flexible and situated nature of a spatial association multiple coding framework. For example, findings from a principle component analysis showed that the SNARC effects in the parity judgment and magnitude classification tasks were placed in two separate components, suggesting unrelated spatial coding mechanisms depending on the task (van Dijck et al., 2012). Moreover, the spatial coding processes underlying spatial associations of numerical magnitudes were found to change depending on task instructions (Georges et al., 2015). In general, considerable heterogeneity across stimulus, task, and participant attributes was documented in a meta-analysis (Wood et al., 2008). Moreover, the replicated dissociation between SNARC effects for number and order information is in line with the taxonomy proposed to disentangle the multitude of mechanisms involved in spatial associations (Patro et al., 2014; Cipora et al., 2015a,b). Interestingly, in right-to-left reading native Hebrew participants, reversed SNARC effects akin to the month-performance during anodal tDCS were previously obtained for sequential stimuli (but not when the magnitude dimension was emphasized in the task instruction; Shaki and Gevers, 2011). However, also in native Hebrew participants, a regular left-to-right SNARC effect for numbers was finally observed when parity-space response mappings were tested on separate days (Zohar-Shai et al., 2017). At large, this result could imply mutual interactions between markedness-based codes (parity-space association) and the active coding strategy for spatial-metric associations. Moreover, the findings by Zohar-Shai et al. (2017) also reiterate the crucial influence of seemingly trivial task design parameters, i.e., counterbalanced key assignments.

By drawing on the linguistic structure of the sequence comparison task, a relatively plausible (and testable) account was proposed by first assuming that multiple mechanisms can be dominant when generating spatial association for any concept. Loosely, this proposal also reflects the earliest models of numerical magnitude representations (e.g., the triple-code model) that assumed a verbal, visual spatial, and analog representation (Dehaene et al., 2003) and the proposal is also consistent with the traditional dual-code assumption for mental representations in general (Paivio, 1986). In greater detail, the markedness property describes the formal requirements for identification of the default member of verbal opposite pair (Nuerk et al., 2004; Proctor and Cho, 2006). A general compatibility effect then consists in better task performance in cases where either default or non-default members are present in both dimensions of a task.

### Polarity asymmetry of tDCS effects in the cognitive domain

The combined results also demonstrate the asymmetry of polarity-specific tDCS effects in the cognitive domain (Jacobson et al., 2012): For numerical stimuli, we found that the effect size for anodal stimulation was low (*d* = 0.2), and the effect turned significant only for the least conservative test across all available data from *N* = 72 healthy participants. Here it should be noted that the three included studies used different number ranges and classification tasks (1–9 in parity judgment or 1–5 in magnitude comparison tasks) and number notations (1–5 or “eins”-“fünf,” magnitude comparison tasks). The mechanisms beyond producing spatial associations for these different number stimuli and tasks are likely to differ (Georges et al., in press). The results of our joint analysis can only suggest that the systematic interactions of these range- and task-effects are (at best) only marginally pronounced in modulating the effect of anodal tDCS on spatial-numerical associations and they are thus less decisive given the statistically non-significant interaction effects. By analysing data drawn from three experiments, the current result shows that anodal tDCS of the left prefrontal cortex can increase the inclination of SNARC effects for numerical stimuli, but the effect size of this stimulation effect is small.

In contrast, the effect of cathodal tDCS on numerical stimuli was relatively effective in previous observations (*d* = 0.5; Schroeder et al., 2016). This pattern of results is remarkably opposite to the typical observation that cathodal tDCS was less effective for modulating cognition (Jacobson et al., 2012; Pirulli et al., 2014). Furthermore, DC polarity-specific effects appeared rather linear and symmetric at physiological level in motor cortex studies showing excitability changes (Priori et al., 1998; Nitsche and Paulus, 2000) and also in *in vitro* studies (e.g., Bikson et al., 2004). The observed patterns suggest that polarity asymmetries are more likely in cognitive tasks and that they can also render the cathodal stimulation more effective than the anodal stimulation (Schroeder and Plewnia, 2016). However, the sources of polarity asymmetry and other non-linear effects of tDCS such as current intensity and individual differences (Batsikadze et al., 2013; Benwell et al., 2015) are currently unknown and must be scrutinized systematically in future research. Finally, the results nicely illustrate that there is no binary outcome of tDCS, but that rather different mechanisms are available for producing spatial associations, which makes the stimulation more efficacious in certain cases.

### Limitations and future directions

The conclusions of this study must be accompanied by some caveats as some further limitations exist. First, given the non-significant positive coefficient in the month sequence during anodal tDCS, it is not clear whether anodal tDCS reverses or abolishes spatial associations of non-numerical ordinal sequences generally^4^. We believe that a reversal better describes the mechanism due to the markedness correspondence account, the significantly positive coefficient in our previous study, the positive sign of the coefficient in this study, and the dissociation with numerical symbols. In future research, it may prove fruitful to study larger stimulus ranges (e.g., longer ranges of sequence (months, letters) during anodal tDCS or single-digit numbers (1–9) during cathodal tDCS).

Secondly, as stimulus attributes and ranges may affect SNARC results, future study designs should ensure that different stimulus ranges in within-subject (within-session) experiments comprise the same numbers of items. This was not the case for the present experiments with different weekday, number, and month name ranges. Given that switching between number ranges may induce different cognitive strategies (Abrahamse et al., 2016; Huber et al., 2016), such manipulations may be studied with care.

Discrepancies in the present results may have been influenced as well by the results during the sham condition. Unlike other reports, we did not observe significantly negative SNARC effects for numbers (and for weekdays) with the 1–5 range without any stimulation (e.g., Dehaene et al., 1993; Fias et al., 1996). It should be acknowledged as well that internal consistency and reliability of the SNARC effect can be medium to low (Cipora and Wood, 2017, for simulations; Cipora and Nuerk, 2013; Viarouge et al., 2014; Georges et al., 2016; for estimates of reliability from 0.27 to 0.70), which may also influence differences between two tDCS conditions.

Several interesting tDCS parameters have not been explored with the present cognitive effects (see Schroeder et al., 2017a, for a review of different tDCS parameters in stimulation studies). It is currently unknown whether changes in intensity or electrode configuration (such as a bilateral vs. extracephalic return electrode configuration) would lead to comparable stimulation outcomes. Moreover, there are no definite data on right-hemispheric tDCS. Also here, the data of Di Rosa et al. (2017) are interesting, because they show dissociations between parity-space and number-space associations in a parietal-supraorbital configuration, independent of cortical hemisphere. However, a possible effect of laterality was not significant in their study and the electrode configuration targeted entirely different areas than the prefrontal-extracephalic configuration used in the current and in our previous studies.

In sum, we view our study as a starting point, which shows at least that the neurocognitive mechanisms underlying the SNARC seem to be not as simple as often assumed. However, because these mechanisms and the codes involved may depend on task, stimuli, participants, and stimulation parameter, we wish to acknowledge that much more research is needed to explore the generality and specificities of associations of space and different cardinal or ordinal metrics.

## Summary

We report mixed evidence for switching between spatial associations from prefrontal anodal tDCS and document positive and negative results within the same group of participants with different non-numerical stimuli. A conceptual replication demonstrates the possibility to modulate spatial associations of a non-numerical sequence (month names) by administration of anodal tDCS to the left prefrontal cortex. In another sequence of weekdays, the manipulation was not successful. The mixed evidence is best accounted for by unexplored systematic variations in study design, individual differences, or task contagion. Results are compatible with the previously proposed model of markedness correspondence (Schroeder et al., 2017b) which accounts for the observed switching between stimulus-response compatibility effects due to markedness processing of target stimuli during anodal tDCS of the left prefrontal cortex. In the proposed multiple-coding framework, spatial-metric associations can result from various verbal and non-verbal simulations whose parameters may be selectively malleable by different tDCS configurations.

## Author contributions

PS, H-CN, and CP designed research. PS performed research and ran analyses. PS wrote first draft and all authors commented and contributed to the final draft.

### Conflict of interest statement

The authors declare that the research was conducted in the absence of any commercial or financial relationships that could be construed as a potential conflict of interest.

## Footnotes

^1^In further explorative analyses, we also modeled SNARC effects in terms of standardized regression weights and categorical compatibility effects. Results from these procedures essentially reproduced the reported results: There were significant differences between SNARC effects for month stimuli during sham vs. anodal tDCS, but not for number and weekday stimuli. For full transparency, all values and tests are reported in the Supplementary Tables ST1 and ST2. A table reporting RTs and error rates for all target-response combinations is provided in Supplementary Table ST3.

^2^Repeated testing of the same participants with different sequences were treated as separate groups of participants in this analysis. Because we did not observe substantial construct validity between spatial associations in different sequences in a larger study (Schroeder et al., in press), we believe that in this case the benefits of easier presentation would outweigh the consequence of overestimated error variance.

^3^Alternatively, this effect may also reflect an induction of regular SNARC effects for numbers by anodal tDCS, since the SNARC effect for numbers was not significantly different from zero during the sham condition (but see also Supplementary Analysis SA2 for Bayes Factors). We wish to thank Reviewer 3 for pointing to this possibility.

^4^In principle, to draw conclusions from insignificant hypothesis test results, Bayesian analyses can be performed. For the reversed SNARC effect with month stimuli during anodal tDCS, we obtained BF = 1.155. Thus, the posterior probability of the null hypothesis was 46.4 % (and the complimentary probability for the alternative hypothesis 53.6 %). Thus, also the Bayesian result presents nothing but anecdotal evidence in the direction of the alternative hypothesis (reversal), on which no strong conclusions can be based on. See SA2 in the supplementary materials for more details on this approach.
