# Supplementary material for: Switching between Multiple Codes of SNARC-Like Associations: Two Conceptual Replication Attempts with Anodal tDCS in Sham-Controlled Cross-Over Design
Source: Front Neurosci. 2017 Nov 23;11:654. doi: 10.3389/fnins.2017.00654 (PMC5703834; doi:10.3389/fnins.2017.00654)
Supplement: Supplementary file 1 [file Table1.DOCX]

Supplementary Material

Switching Between Spatial Associations with Anodal tDCS: Two Conceptual Replications in Sham-Controlled Cross-Over Design

Philipp Alexander Schroeder*, Hans-Christoph Nuerk, Christian Plewnia

*** Correspondence:** Corresponding Author: [philipp.schroeder@uni-tuebingen.de](mailto:philipp.schroeder@uni-tuebingen.de)

**Supplementary Table ST1**. Regression coefficients of spatial associations. Unstandardized b regression coefficients indicate response hand advantage increases (SNARC and SNARC-like effects) with single position/magnitude increments in the corresponding sequence (dRT/position[ms]), whereas standardized β coefficients correct for individual variance. Pseudo-position bins (1-5) were assigned to all sequences. For the month series, consecutive month names were aggregated to single position bins (e.g., January + February = position “1”). All regression coefficients were individually extracted and the resulting group-level mean was tested with one-sample t-tests against zero (cf. Fias, Brysbaert, Geypens, & D’Ydewalle, 1996).

|  | **sham** | | | |  | **anodal tDCS** | | | |  | ***t*-test** | |  | | ***t-*test beta** | | |
| --- | --- | --- | --- | --- | --- | --- | --- | --- | --- | --- | --- | --- | --- | --- | --- | --- | --- |
| **sequence** | b | *p* | β | *p* |  | b | *p* | β | *p* |  | *t* | *p* | |  | | *t* | *p* |
| numbers (‘one’-‘five’) | -4.157 | .386 | -.029 | .817 |  | -6.740 | .032 | -.280 | .025 |  | 0.53 | .533 | |  | | 1.61 | .120 |
| weekdays (Mo-Fr) | -3.087 | .462 | -.263 | .049 |  | -8.746 | .058 | -.204 | .089 |  | 0.75 | .460 | |  | | 0.31 | .762 |
| months (Jan-Dec) | -7.445 | .057 | -.190 | .135 |  | +4.217 | .175 | +.069 | .458 |  | 2.68 | .013 | |  | | 2.20 | .038 |

**Supplementary Table ST2.** Compatibility Effects (CEs). CEs were computed by calculating the RT difference between SNARC congruent and SNARC-incongruent trials, defined as trial combinations of left + small / early, right + large / late (SNARC-congruent), and vice versa.

|  | **sham** | |  | **anodal tDCS** | | | ***t*-test** | | | | |  | |
| --- | --- | --- | --- | --- | --- | --- | --- | --- | --- | --- | --- | --- | --- |
| **sequence** | CE [ms] | *p* |  | CE [ms] | *p* |  | |  |  | *t* | *p* | |  |
| numbers (‘one’-‘five’) | 12.6 | .179 |  | 18.6 | .003 |  | |  |  | 0.53 | .514 | |  |
| weekdays (Mo-Fr) | 15.1 | .012 |  | 16.7 | .059 |  | |  |  | 0.75 | .891 | |  |
| months (Jan-Dec) | 10.3 | .126 |  | -4.5 | .403 |  | |  |  | 2.68 | .047 | |  |

**Supplementary Table ST3.** Median response times (RTs) and error rates for all target-response combinations in the three tasks and two stimulation conditions.

|  | **sham** | | | |  | **anodal tDCS** | | | | |  |
| --- | --- | --- | --- | --- | --- | --- | --- | --- | --- | --- | --- |
| **sequence** | RT left [ms] | RT right [ms] | Error left [%] | Error right [%] |  | RT left [ms] | RT right [ms] | Error left [%] | Error right [%] |  | |
| numbers (‘one’-‘five’) |  |  |  |  |  |  |  |  |  |  | |
| One | 484 | 480 | 6.3 | 3.3 |  | 477 | 481 | 2.9 | 1.6 |  | |
| Two | 509 | 499 | 6.2 | 7.1 |  | 503 | 499 | 5.7 | 5.4 |  | |
| Four | 539 | 499 | 9.5 | 10.4 |  | 534 | 510 | 12.0 | 7.7 |  | |
| Five | 502 | 493 | 3.8 | 2.1 |  | 507 | 487 | 2.7 | 4.7 |  | |
| weekdays (Mo-Fr) |  |  |  |  |  |  |  |  |  |  | |
| Monday | 512 | 499 | 3.9 | 6.5 |  | 520 | 515 | 4.4 | 6.0 |  | |
| Tuesday | 538 | 523 | 10.5 | 6.1 |  | 543 | 543 | 9.2 | 6.0 |  | |
| Thursday | 545 | 515 | 5.3 | 6.9 |  | 554 | 513 | 5.9 | 6.2 |  | |
| Friday | 515 | 494 | 7.4 | 7.3 |  | 529 | 501 | 5.0 | 9.6 |  | |
| months (Jan-Dec) |  |  |  |  |  |  |  |  |  |  | |
| January | 494 | 503 | 7.6 | 5.8 |  | 512 | 508 | 6.4 | 6.5 |  | |
| February | 531 | 524 | 10.3 | 10.2 |  | 550 | 518 | 9.6 | 7.5 |  | |
| March | 486 | 483 | 3.3 | 5.8 |  | 503 | 484 | 3.3 | 4.8 |  | |
| April | 520 | 515 | 2.9 | 5.9 |  | 520 | 515 | 4.8 | 3.4 |  | |
| September | 503 | 488 | 4.5 | 4.4 |  | 515 | 509 | 2.9 | 2.6 |  | |
| October | 527 | 491 | 4.6 | 5.7 |  | 511 | 503 | 4.8 | 5.8 |  | |
| November | 524 | 486 | 3.1 | 5.5 |  | 517 | 509 | 5.2 | 2.2 |  | |
| December | 500 | 477 | 3.5 | 3.5 |  | 498 | 495 | 2.9 | 2.4 |  | |

**Supplementary Analysis SA1.** Alternative analysis of main findings without data preprocessing.

Based on a reviewers concern with our data preprocessing strategy, we also submitted directly aggregated data from correct mean RTs to our main analyses. More precisely, the following preprocessing steps were skipped in this type of analysis: All but error trials were considered for RT analysis, including stimulus repetitions, trials following errors, and no outliers were rejected. Globally, this strategy led to considerably smaller percentage of trial rejection (4.6 %) than the more conservative strategy presented in the paper (26.5 %). In this supplementary analysis here, mean correct RTs were aggregated for each stimulus, upon which we calculated dRTs (left-hand – right-hand RT) and then fitted regression coefficients based on the numerical magnitude or sequential position predictor, as described in the main methods section.

The results from this procedure were as follows:

1. Number words. SNARC effects with negative-signed coefficients were significantly different from zero for the performance during anodal tDCS (b = −11.93 ms/bin; *t*(23) = −3.67, *p* = .001), but not during the sham tDCS condition (b = −6.24 ms/bin; *t*(23) = −1.57, *p* = .131). The difference in SNARC effects was not significant (*t*(23) = −1.44, *p* = .163).

2. Monday-to-Friday Sequence Words. SNARC effects with negative-signed coefficients were significantly different from zero for the performance during anodal tDCS (b = −9.74 ms/bin; *t*(23) = −3.07, *p* = .005) and also during the sham tDCS condition (b = −8.09 ms/bin; *t*(23) = −2.41, *p* = .024). The difference in SNARC effects was not significant (*t*(23) = −0.35, *p* = .732).

3. January-to-December Sequence Words. A reversed SNARC effect with positive-signed coefficient was not significant during the anodal tDCS condition (b = +3.05 ms/bin; *t*(23) = 0.91, *p* = .373). The SNARC effect for the same month stimuli was negative-signed during the sham condition (b = −7.45 ms/bin; *t*(23) = −1.71, *p* = .102). In direct comparison of the two stimulation conditions, the effect of anodal tDCS was significant (*t*(23) = 2.49, *p* = .020, *d* = 0.51).

At large, the obtained results from the alternative analysis corroborate our main conclusions and, again, we could not find significant stimulation effects for number and weekday stimuli, but only for month stimuli. Nevertheless, we decided to present this analysis as well, because we agree with the issue that data preprocessing can offer opportunities for questionable research practices (Simmons, Nelson, & Simonsohn, 2011; Gelman & Loken, 2014) and should be transparently addressed, particularly in the case that controversial effects are investigated. Our main conclusions are thus corroborated by alternative data preprocessing strategies as well.

**Supplementary References**

Gelman, A., & Loken, E. "The Statistical Crisis in Science Data-dependent analysis—a “garden of forking paths”—explains why many statistically significant comparisons don't hold up." *American Scientist* 102.6 (2014), 460.

Simmons, J. P., Nelson, L. D., & Simonsohn, U. (2011). False-positive psychology: Undisclosed flexibility in data collection and analysis allows presenting anything as significant. *Psychological science*, *22*(11), 1359-1366.

**Supplementary Analysis SA2.** Bayes Analyses for Inconclusive Null Results

In frequentist statistics, null results can never allow researchers to reject the alternative hypothesis. To overcome this shortcoming, Bayesian statistics allow to quantify the evidence favouring either null or alternative hypothesis. In the current results, two major concerns were not sufficiently addressed by merging data from previous studies: 1) There was no significant SNARC effect for number words during sham tDCS and 2) the significant tDCS modulation of SNARC effect for month words did not lead to a significantly reversed effect. Both observed results are not compatible with previous literature on notation-independent SNARC effects (Nuerk et al., 2004; Nuerk, Wood, & Willmes, 2005) and with the effects of anodal tDCS on non-numerical sequences (Schroeder, Nuerk, & Plewnia, 2017). Thus, we analysed these data also in a Bayesian framework with the open-source software JASP (JASP Team, 2016). The respective analyses yielded Bayes Factors (BF), which can be used to rate the evidence favouring the alternative hypothesis (BF > 1) or the null hypothesis (BF < 1) and compute posterior probabilities. Jeffreys (1961) proposed a BF below 3 (above 1/3) presents anecdotal evidence, 3-10 (1/3-1/10) presents moderate evidence, 10-30 (1/10-1/30) presents strong evidence, and so on, for H1(H0).

For the SNARC effect with number words during sham tDCS, we obtained BF = 0.305. Thus, according to Masson (2011), the posterior probability of the null hypothesis was 76.6 %. Thus, the Bayesian result can only be rated as anecdotal/moderate evidence for the null hypothesis.

For the reversed SNARC effect with month stimuli during anodal tDCS, we obtained BF = 1.155. Thus, the posterior probability of the null hypothesis was 46.4 % (and the complimentary probability for the alternative hypothesis was 53.6 %). Here, also the Bayesian result presents nothing but anecdotal evidence in the direction of the alternative hypothesis.

**Supplementary References**

JASP Team. (2016). JASP (Version 0.7.5.5).

Jeffreys, H. (1961). *Theory of probability* (3rd edn). Oxford, UK: Oxford University Press.

Masson, M. E. J. (2011). A tutorial on a practical Bayesian alternative to null-hypothesis significance testing. *Behavior Research Methods, 43*, 679–690. http://doi.org/10.3758/s13428-010-0049-5

Nuerk, H.-C., Iversen, W., & Willmes, K. (2004). Notational modulation of the SNARC and the MARC (linguistic markedness of response codes) effect. *The Quarterly Journal of Experimental Psychology, 57*(5), 835–63. http://doi.org/10.1080/02724980343000512

Nuerk, H.-C., Wood, G., & Willmes, K. (2005). The universal SNARC effect: the association between number magnitude and space is amodal. *Experimental Psychology, 52*(3), 187–94. http://doi.org/10.1027/1618-3169.52.3.187

Schroeder, P. A., Nuerk, H.-C., & Plewnia, C. (2017). Prefrontal neuromodulation reverses spatial associations of non-numerical sequences, but not numbers. *Biological Psychology, 128*(February), 39–49. http://doi.org/10.1016/j.biopsycho.2017.07.008
